# Leads with the Cut Proximal Ends Migrated into the Heart and Vasculature: A Rare Phenomenon among 3847 Lead Extraction Procedures

**DOI:** 10.3390/jcm13092602

**Published:** 2024-04-29

**Authors:** Andrzej Kutarski, Wojciech Jacheć, Radosław Pietura, Paweł Stefańczyk, Jarosław Kosior, Marek Czajkowski, Sebastian Sawonik, Łukasz Tułecki, Dorota Nowosielecka

**Affiliations:** 1 Department of Cardiology, Medical University of Lublin, 20-093 Lublin, Poland; a_kutarski@yahoo.com (A.K.);; 2 2nd Department of Cardiology, Faculty of Medical Sciences in Zabrze, Medical University of Silesia, 40-055 Katowice, Poland; 3 Department of Radiography, Medical University of Lublin, 20-093 Lublin, Poland; 4 Department of Cardiology, The Pope John Paul II Province Hospital of Zamosc, 22-400 Zamosc, Poland; 5 Department of Cardiology, Masovian Specialistic Hospital of Radom, 26-617 Radom, Poland; 6 Department of Cardiac Surgery, Medical University of Lublin, 20-093 Lublin, Poland; 7 Department of Cardiac Surgery, The Pope John Paul II Province Hospital of Zamosc, 22-400 Zamosc, Poland

**Keywords:** leads with ends migrated into the cardiovascular system, fractured leads, extraction of migrated leads, management of fractured leads

## Abstract

**Background**: The study aimed to describe the phenomenon of leads migrated (MPLE) into the cardiovascular system (CVS). **Methods**: Retrospective analysis of 3847 transvenous lead extractions (TLE). **Results**: Over a 17-year period, 72 (1.87%) MPLEs (median dwell time 137.5 months) were extracted, which included mainly ventricular leads (56.94%). Overall, 68.06% of MPLEs had their cut proximal ends in the venous system. Most of them were pacing (95.83%) and passive fixation (98.61%) leads. Independent risk factors for MPLE included abandoned leads (OR = 8.473; *p* < 0.001) and leads located on both sides of the chest (2.981; *p* = 0.045). The higher NYHA class lowered the probability of MPLE (OR = 0.380; *p* < 0.001). Procedure complexity was higher in the MPLE group (procedure duration, unexpected procedure difficulties, use of additional (advanced) tools and alternative venous approach). There were no more major complications in the MPLE group, but the rate of procedural success was lower due to more frequent retention of non-removable lead fragments. Extraction of MPLEs did not influence long-term survival. **Conclusions**: 1. Extraction of leads with MPLE is rare among other TLE procedures (1.9%), 2. risk factors include abandoned leads and presence of leads on both sides of the chest but a higher NYHA class lowers the probability of MPLE, 3. complexity of MPLE extraction is higher regarding procedure duration, unexpected procedure difficulties, use of advanced tools and techniques but rates of major complications are comparable, and 4. extraction of MPLEs did not influence long-term survival.

## 1. Introduction

Incorrect fixation of the retained cut leads, lead fractures due to ligature that is too tight and improper subclavian vein puncture with secondary crush syndrome can make the proximal end of the lead slip into the CVS and move further [1,2,3,4,5,6,7,8,9,10,11,12,13,14,15].

The migration of the cut proximal lead end (MPLE) into the subclavian or anonymous vein [3,7,10,11,12,13], or even superior vena cava [1] ends up with loops, which in turn pass through the tricuspid valve to the right ventricle, triggering tricuspid valve dysfunction [1] and ventricular arrhythmias [6,16]. Sometimes, free MPLEs float via the right heart cavities into the pulmonary artery [1,2,3,5,6,7,8], causing pulmonary embolism [1,12]. For these reasons, MPLEs in the CVS become a potential source of serious secondary consequences and a class 1 indication (lead with an ending in the CVS, which may pose an immediate threat to the patient if left in place, life threatening arrhythmias secondary to retained lead or lead fragment) or class 2b indication (lead which may pose a potential future threat to the patient if left in place) for lead extraction according to the guidelines of the Heart Rhythm Society (HRS) [17,18]. This phenomenon has been described in numerous case reports [4,5,6,7,8,9,10,11,12,13,14,15], case series [3] and few publications [1,2,16] and mentioned in the guidelines [19]. Having a large, computerized database of extraction procedures, we decided to perform a deeper analysis of this phenomenon.

### 1.1. Aim of the Study

The aim of our study was to analyse migration of the cut proximal lead ends (MPLEs) into the cardiovascular system (CVS) in the last 17 years and to determine its frequency, type and age of migrated leads, location of MPLEs, risk factors for lead migration dependent on the patient and the CIED system, predictors of major complications or increased procedure complexity and finally, to describe complexity and complications of migrant lead extraction, and its influence on long-term outcomes.

### 1.2. What Is New?

Spontaneous conductor fractures and insulation breaks of the intracardiac leads near their venous entry or incorrect fixation of the retained cut leads sometimes cause the proximal end of the fractured lead to slide into the veins, with potential subsequent looping in the heart and secondary complications. So far, this topic has not been extensively studied. As it is a relatively uncommon finding, it has been described in a vast number of case studies only. To the best of our knowledge, this is the first comprehensive description of the phenomenon, its risk factors, and management of the cut proximal lead ends in the cardiovascular system.

## 2. Methods

### 2.1. Study Population

All transvenous lead extraction procedures (TLE) performed between March 2006 and March 2023 at three high-volume centres were reviewed. Patient clinical characteristics, CIED system and history of pacing, data on targeted leads, TLE complexity, efficacy and outcomes were retrospectively analysed using our computerized database. The study population consisted of 3847 patients, aged 5–99 years, the mean age was 66.02 years, and 38.01% were women.

### 2.2. Lead Extraction Procedure

Indications for lead extraction, effectiveness and complications of the procedure were defined according to the recent recommendations (2009 and 2017 HRS consensus and 2018 EHRA expert consensus statement) [17,18,19]. The TLE was expressed as the rate of procedural success and clinical success [17,18,19]. The complications of TLE were also defined as major complications that were life threatening, resulted in significant or permanent health disability or death, or required surgical intervention [17,18,19]. 

#### 2.2.1. Procedure Complexity

Procedure complexity was expressed as whole lead extraction time (sheath-to-sheath time) and average time of single lead extraction (sheath-to-sheath/number of removed leads), and use of second line tools and advanced tools [20,21]. The third (new) complexity marker was The Complex Indicator of the Difficulty of the TLE (CID TLE) which included global sheath-to-sheath time for extraction of all leads >20 min (2 points), average duration of single lead extraction (sheath-to-sheath time) >12 min (2 points) and use of metal sheaths or Evolution/TightRail, alternative approach or lasso-catheters or basket catheters (one point for each). The sum of points was the value of CID-TLE [20].

#### 2.2.2. Unexpected Technical Problems during TLE

They covered all situations that increased procedure complexity but were not complications. They included blockage in the lead venous entry/subclavian region preventing advancement of a polypropylene catheter into the subclavian vein, Byrd dilator collapse/fracture, lead-on-lead adhesion, necessity of using an alternative approach, loss of fractured lead fragment when the main part of the lead was dissected and removed but both free ends were retained, the mobile lead fragment which floated usually into the pulmonary circulation, and displacement of functional leads [21].

#### 2.2.3. Procedure Information

We utilized a stepwise approach in all patients. Standard stylets or locking stylets (Liberator Locking Stylet, Cook Medical Inc., Bloomington, IN, USA) were used, the latter ones for extraction of the oldest leads with a high estimated risk of fracture. We usually started with non-powered mechanical telescoping polypropylene sheaths (Byrd Dilator Sheaths, Cook Medical Inc., USA) of all lengths and sizes. The second-line tools included powered mechanical sheath systems (Evolution Mechanical Dilator Sheath, Cook Medical, Bloomington, IN, USA; TightRail Rotating Dilator Sheath, Phillips, Colorado Springs, CO, USA) or metal sheaths if the obstacle was encountered in the extracted lead venous entry region. A combined approach, using two or more different (jugular, subclavian, femoral) access sites, was selected when conventional methods were presumed ineffective (proximal lead ends in the cardiovascular space or in case of lead fracture during extraction) [1,2,3,11,12,13,15]. 

#### 2.2.4. Leads with Proximal Ends Migrated into the Cardiovascular System—Definitions

Leads with their cut or spontaneously broken proximal ends migrated into the cardiovascular system, for various reasons and via multiple mechanisms, were defined as fractured leads in the lead implant vein when their proximal ends slipped into the CVS and migrated from the subclavian vein into the superior vena cava, right atrium, right ventricle into the pulmonary artery or rarely another vein while the distal ends (tips of the lead) remained where the lead had been implanted [1,2,3,7,8,12,13,15].

#### 2.2.5. Extraction of Leads with Their Proximal Ends Migrated into the Cardiovascular System

We always attempted to remove all leads with their cut or broken proximal ends migrated into the cardiovascular system (CVS). These leads were regarded not only as abandoned non-functional leads, but also as a potential source of adverse consequences of the migrant proximal lead end (thrombosis, venous occlusion, arrhythmias) as well as adverse or potentially adverse consequences of lead looping in the heart (TV dysfunction, arrhythmias). One of these consequences is accelerated adhesion of the lead loop to the venous wall, which may significantly hinder the extraction of such leads in case of future infectious complications [1,2,7,8,11,14,16]. For this reason, we treated all such leads as “leads which may pose an immediate threat to the patient if left in place or leads which may pose a potential future threat to the patient if left in place” [17,18].

**Figure 1:**
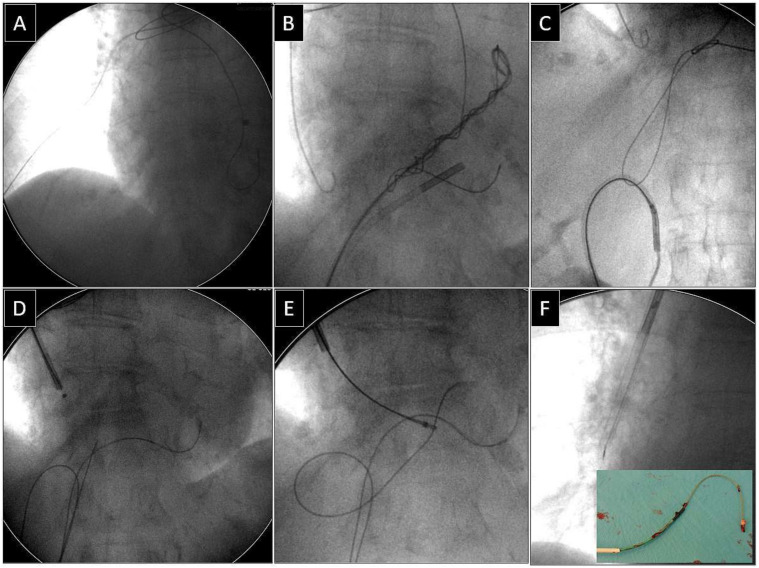
Extraction of a fractured old model unipolar passive fixation ventricular lead with its proximal end in the branch of the right pulmonary artery. The lead without a graspable proximal end (**A**). A “spaghetti twisting technique” was used to catch the lead with a pig-tail catheter. After simple winding and pulling on the lead the end of the lead was partially withdrawn from the pulmonary artery (**B**). The technique of pulling down the lead with the loop had to be used (via the femoral approach) (**C**,**D**). The proximal end of the lead was grasped with a jugular lasso catheter (**E**). Conventional lead dissection with polypropylene sheaths proved to be successful (**F**). Removed lead on the table (**F**). An example of using a combined approach (jugular and femoral).

**Figure 2:**
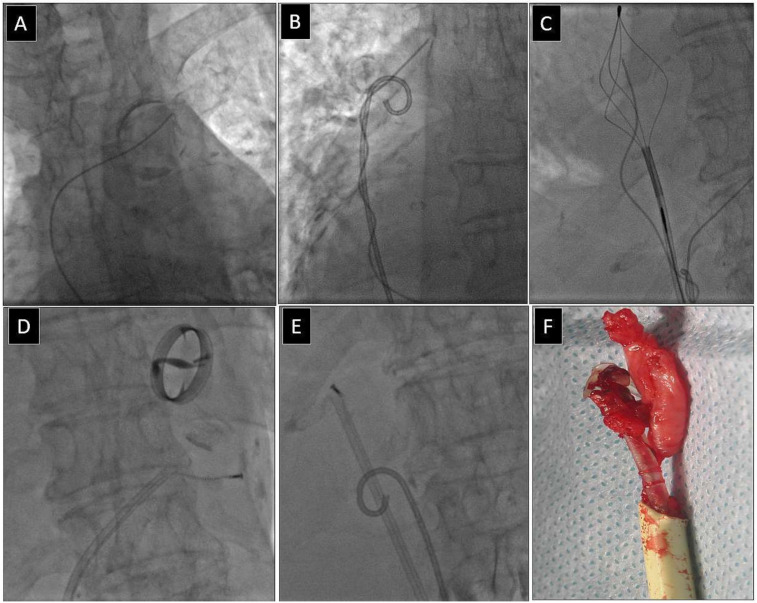
Extraction of a fractured old model unipolar passive fixation ventricular lead with its proximal end in the anonymous vein. The lead without a graspable proximal end (**A**). Again, a “spaghetti twisting technique” was used with a pig-tail catheter. After winding and pulling on the lead, the proximal end of the fractured lead was pulled to the right atrium. (**B**). The end of the fractured lead was grasped with a basket catheter inside the right atrium (**C**). Conventional lead dissection with a polypropylene sheath (**D**,**E**). The clamped basket catheter extends the fractured lead and allows usual lead dissection in the intraventricular section. Extracted lead on the table (**F**). An example of using a femoral approach for fractured lead extraction.

**Figure 3:**
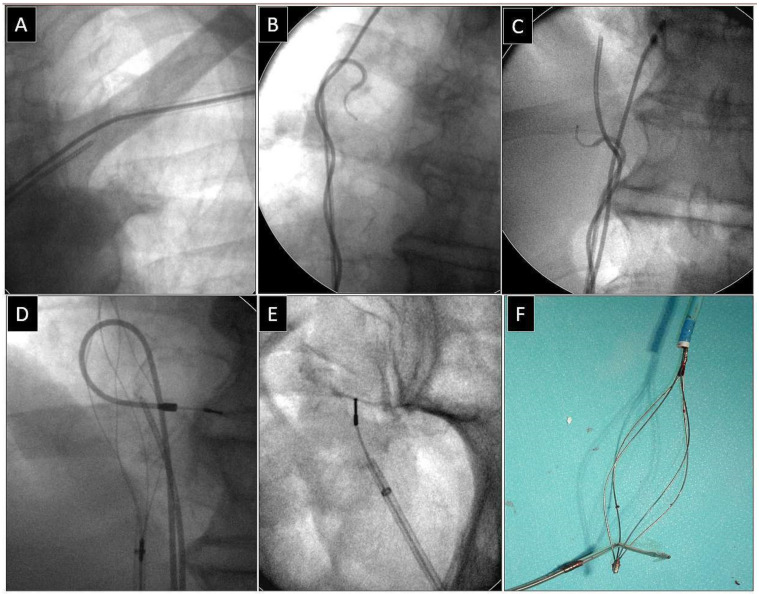
Extraction of a fractured bipolar passive fixation ventricular lead with its proximal end in the subclavian vein. Similarly, the lead without a graspable proximal end (**A**). Also, in this case the “spaghetti twisting technique” using a pig-tail catheter was found to be effective. After winding and pulling on the lead, the proximal end of the fractured lead was pulled to the right atrium. (**B**,**C**). The end of the fractured lead was grasped with a basket catheter in the low right atrium (**D**). The clamped basket catheter extended the fractured lead. Conventional counter-traction was enough to remove the lead (**E**,**F**). Extracted broken lead on the table (**F**). A pig-tail catheter and “spaghetti twisting technique” were used to make the proximal end accessible from a femoral approach. An example of using a femoral approach for fractured lead extraction.

Depending on the location of the proximal end of the fractured and displaced lead, we tried to retrieve it with a lasso (Figure 1) or basket catheter (Figure 2 and Figure 3) using the femoral approach (Figure 2 and Figure 3) or, if other leads were planned for extraction, we sometimes tried to use the subclavian access re-established after extraction of another lead [1,2]. Jugular access was used less frequently (Figure 1). After firmly grasping the proximal end of the migrated lead, the lasso or basket catheter played a role of an extension (Figure 1, Figure 2 and Figure 3) of the fractured lead and we performed lead dissection until the lead was removed using polypropylene sheaths (Figure 1 and Figure 2) or 16F silicone catheters (Curved Femoral Introducer Sheath Set 16 Fr, LR-CSS16) with a bevelled end for rotational dilation [1,2,11,12]. Mechanical rotational tools were rarely used, as most of the procedures had been performed before these tools became available on the market. We rarely used the Needle’s Eye Snare© because other techniques proved to be more effective. When the proximal end of the fractured migrated lead could not be grasped, we released it from the fibrous tissue by wrapping the lead around a pig-tail catheter (“spaghetti twisting technique”) [1,2,7,8,13] (Figure 2 and Figure 3) or by using the loop made of a guidewire and a lasso catheter or basket catheter placed over the removed lead and pulled (Figure 1) [1,2,5,11,12,15], if the “spaghetti twisting technique” was entirely ineffective. 

We did not use sheaths equipped with laser energy. In the last 17 years, the organization of lead extraction has evolved from procedures performed in the electrophysiology laboratory using intravenous analgesia/sedation to procedures performed in the hybrid room only under general anaesthesia [22,23]. Over the last 7 years, the core extraction team has consisted of the same highly experienced extractor (now frequently serving as a proctor), experienced echocardiographer and cardiac surgeon experienced in the treatment of TLE complications [23,24].

### 2.3. Dataset and Statistical Methods

#### 2.3.1. Creation of Subgroups for Future Analysis

Based on the analysis of 3847 extraction procedures, they were divided into two subgroups: 1. transvenous extraction of leads with their cut or broken proximal ends spontaneously migrated (MPLE) into the cardiovascular system (CVS) (72 procedures), 2. transvenous extraction of leads without spontaneous migration into the CVS (control group, 3775 procedures).

#### 2.3.2. Statistical Analysis

Due to nonparametric distribution, all continuous variables are presented as the median and lower and upper quartile (Q1–Q3). The categorical variables are presented as counts and percentages. The significance of differences between the groups was determined using the nonparametric Chi^2^ test with Yates correction or the unpaired Mann–Whitney U test, as appropriate. Univariable and multivariable regression was used to identify the factors that influenced the probability of MPLE. Variables with *p* values less than 0.1 on univariable analysis were entered into the multivariable model. Survival of the patients was compared using the log rank test. A *p*-value less than 0.05 was considered statistically significant. Statistical analysis was performed with Statistica 13.3 (TIBCO Software Inc. Tulsa, OK, USA).

### 2.4. Approval of the Bioethics Committee

All patients provided written informed consent to undergo TLE and to have anonymous data from their medical records used for research purposes. The research methodology was approved by the Bioethics Committee at the Regional Chamber of Physicians in Lublin no. 288/2018/KB/VII (approval date: 27 November 2018). The study was carried out in accordance with the ethical standards of the 1964 Declaration of Helsinki.

## 3. Results

Removal of leads with their proximal ends spontaneously migrated into the cardiovascular system (MPLE) is relatively rare among other transvenous lead extraction procedures (1.87%). Retrospective analysis showed that most of these procedures have been performed ≥10 years earlier. (Table 1).

When lead removal became available at our facility 17 years ago, it opened up the possibility of admitting patients who could not be admitted before. After performing all “overdue” procedures, the number of new referrals stabilized at about 0.5%. 

Most of the leads spontaneously migrated into the cardiovascular system (MPLE) were ventricular leads (56.94%). Proximal ends of such leads were in the vasculature (subclavian, anonymous and vena cava) (68.06%), and leads in the right atrial location were rare (2.78%) but not in the pulmonary artery (9.72%). Most of such leads were pacing leads (95.83%) and passive fixation leads (98.61%). The median of dwell time of MPLEs was 137.5 months and ranged from 17.24 to 376.4 months (Table 2).

Patients with extraction of MPLEs were younger at the first system implantation (50.50 vs. 61.00 years), less likely to have ischaemic heart disease (37.50 vs. 56.24), non-significantly more often presenting with systemic infection (29.17 vs. 21.48%) and (obviously) much more frequently with threatening/potentially threatening leads as the main indication for TLE (31.94 vs. 2.81%) as compared to those without extraction of MPLEs (Table 3).

Analysis of system- and procedure-related risk factors for increased procedure complexity and major complications showed that patients with extraction of MPLEs had longer lead implant duration before TLE (144.0 vs. 86.04 months) and global lead dwell time before TLE (22.63 vs. 12.00 years), more frequent presence of abandoned leads (68.06% vs. 9.75%), presence of unnecessary (large) lead loops in the heart (54.17% vs. 3.79%), higher number of leads in the heart before TLE (2.57 vs. 1.94), more frequently multiple leads (4 and >4) in the heart (22.22% vs. 2.70%), leads on both sides of the chest (25.00% vs. 2.36%) and more procedures before lead extraction (2.82 vs. 1.84) and two or more CIED-related procedures before TLE (88.89 vs. 52.16%) (Table 4).

Although none of the prognostic scores/calculators of increased procedure complexity included the presence of leads with MPLEs, all scores such as SAFETY score [risk of MC] [25], EROS score [risk of MC] [26], MB score [need for advanced tools] [27], LED index [predicted fluoroscopy time] [28], Advanced TLE scale [need for advanced TLE techniques] [29,30] and LECOM score [predicted procedure complexity] [20] indicated a significantly higher chance of a difficult and complicated procedure in patients with MPLEs (Table 4).

Analysis of potential procedure-related risk factors for major complications and procedure complexity showed a higher number of extracted leads per patient (2 (1–3) vs. 2 (1–2)), more frequent extraction of abandoned leads (65.28% vs. 8.90%), extraction of old model UP leads (41.76% vs. 8.45%), extraction of passive fixation leads (97.22% vs. 57.22%), extraction of leads with abnormal loops in the heart (52.78% vs. 3.87%) in the MPLE group. Similarly, implant duration was significantly longer: longest lead dwell time (137.5 vs. 84.00 months) and longer average targeted lead implant duration per patient (9.96 vs. 6.86 years).

The comparative analysis showed that in patients with MPLEs, extraction complexity was higher than in the control group because the procedure duration expressed as global lead dissection time (55.00 vs. 9.00 min) and time of single lead extraction were significantly longer (50.00 vs. 4.50 min). Similarly, unexpected procedure difficulties (technical problems) were more frequent (25.00% vs. 5.72%) in patients with MPLE extraction. The use of additional (advanced) tools was more frequent during such procedures in comparison with TLE in the control group: lasso catheters/snares/(52.78% vs. 2.94%), basket catheters (45.83% vs. 0.58%), loops to releasing the end of the lead (70.83% vs. 0.11%), pig-tail catheters (11.11% vs. 0.42%) and alternative approach (79.17% vs. 1.85%). It is worth mentioning that there is another phenomenon that occurs during lead extraction: extracted lead fracture. For this reason, these types of tools were used in the control group, too, but rarely. The complexity of the extraction procedure is well reflected in the CID-TLE which is also called the “retrospective TLE combined difficulty score” (including dilatation time, use of second line tools, advanced tools and advanced techniques): (3.85 vs. 0.49 points) (Table 5).

The two study groups did not differ in the rate of major complications (2.78% vs. 2.01%), but they did in the rate of clinical success (91.67% vs. 98.09%) and procedural success (87.50% vs. 95.39%). This can be easily explained by the more frequent retention of a non-removable fragment of the lead (12.50% vs. 4.00%), which is mainly due to the global implant duration in patients with MPLEs. Mortality after the extraction procedure was similar in the two groups. (Table 5).

Multivariable regression analysis showed that abandoned leads (OR = 8.473; *p* < 0.001) and leads on both sides of the chest (2.981; *p* = 0.045) were independent risk factors for MPLE. Patients with a higher NYHA FC class had a lower probability of MPLE (OR = 0.380; *p* < 0.001) (Table 6).

## 4. Discussion

This study describes a rare and increasingly rare phenomenon of the cut or broken proximal end of the lead migrated into the cardiovascular system. The complication is causally related to implantation errors (too parasternal puncture and crush syndrome, and too strong clamping of the ligature fixing the lead), cutting the connector of the abandoned lead and leaving such leads in the infected generator pocket.

A review of the literature, i.e., multiple case studies [4,5,6,7,8,9,10,11,12,13,14,15,16,17,18,19], two smaller studies [1,2] and case series reports [3,4] including a total of 76 cases [1,2,3,4,5,6,7,8,9,10,11,12,13,14,15] shows that MPLE slightly more often affects ventricular leads (55.5%) and significantly more often pacing leads than ICD leads (single cases only). Of the 76 MPLEs, the proximal end of the fractured lead was located in the venous system (55.3%), pulmonary artery (25.0%) and right ventricle (14.4%), and less often in the right atrium (5.2%). The indications for TLE in these 76 cases were systemic infection or local pocket infection 35 (46.0%), non-infectious indications in 41/76 cases (53.9%) including prophylactic indications 19/76 (25.0%), pacing disturbances 16/76 (21.0%) and interactions with the ICD system 2/76 (2.6%), and ventricular arrhythmias 4/76 (5.3%). In three cases (3.9%), the MPLEs were left in place because they were entirely asymptomatic [6,8].

It is obvious, however, that in the case of the extraction of leads with a shorter stay in the patient’s body, the procedure is likely to be of low complexity, the risk of major complications is negligible, and selected patients even have a chance of being discharged on the same day [30].

The present study showed that extraction of leads with their proximal ends migrated into the CVS was relatively rare among other TLEs (2.5%). The rate of new referrals stabilized at about 0.5%.

Most of the MPLEs were ventricular leads (56,9%) and their proximal ends were in the venous system (68.1%) or in the RV (18.0%), pulmonary artery (9.7%) and in the RA (2.6%). Most of them were pacing leads (95.8%), and passive fixation leads (98.6%), and their dwell time ranged from 17 to 376 months, with a median of 137.5 months.

The patients with MPLEs were younger at the first system implantation and less likely to develop IHD but (obviously) more often, the main indication for TLE was a threatening/potentially threatening lead in comparison with the control group.

The patients with MPLEs had longer implant duration, more frequently abandoned leads, unnecessary (large) lead loops in the heart, more leads in the heart, leads on both sides of the chest and more CIED-related procedures before lead extraction. Lead abandonment, including leads on both sides of the chest significantly increased the risk of MPLE. On the other hand, heart failure expressed as a higher NYHA FC class decreased the probability of this complication.

Although none of the prognostic scores/calculators of increased procedure complexity included the presence of MPLE, all scores indicated a significantly higher chance of a difficult and complicated procedure in such patients.

The level of TLE complexity was higher in the MPLE group than in the control group regarding procedure duration, unexpected technical problems, use of additional (advanced) tools and alternative approach.

There were no more major complications in the MPLE group, but the rate of clinical success and procedural success was lower because of the more frequent retention of a non-removable lead fragment.

Extraction of migrant leads with their cut proximal ends in the heart and vasculature should be included in the TLE training program.

## 5. Conclusions

1. Removal of leads with their cut or broken proximal ends migrated into the heart and vasculature was rare among other extraction procedures (1.87%).
2. Lead abandonment and leads located on both sides of the chest significantly increased the risk of MPLE. Higher NYHA FC class decreased the probability of this complication.
3. Procedure complexity in patients with MPLEs is higher in regards to procedure duration, unexpected procedure difficulties, use of advanced tools and techniques but there are no more major complications.
4. Extraction of leads migrated to the heart and vasculature had no effect on long-term survival.

## 6. Study Limitations

This study has some limitations. It describes the experience of the same first operator serving usually as a proctor at three facilities. Data were collected prospectively but analysed retrospectively. All procedures were performed using all kinds of mechanical systems but not laser powered sheaths. An important limitation of the present study is the selection of patients. It would be ideal to assess the incidence of a migrant lead using a very large database of all implantations and patient histories collected for 10–20 years. Our research is limited to a specific population, i.e., patients referred to our facility as the reference TLE centre (15–10 years ago), and from other high- and mid- volume centres. This population does not reflect all patients with implanted CIEDs. We do not know anything about patients with migrant leads and their fate who were not referred for TLE.

A certain minor limitation of the present study is the fact that some of the case studies mentioned in the discussion section were previously described based on our material by investigators from other national centres. The study analyses a very large population of patients undergoing TLE with an implant duration longer than in many recent studies because our centre for many years was an unofficial reference centre and we were receiving the most difficult patients in the country. This explains the rate of major complications and a lower rate of radiographic success. Our experiences should be of interest to all those who will face extractions of old passive-fixation leads and management of less frequent lead-related permanent pacing complications.

## Figures and Tables

**Table 1:** Leads with their proximal ends migrated into the cardiovascular system in the last 16 years.

Four Similar Consecutive Periods	Years	Number of TLE Procedures	Extraction of Leads with Their Proximal Ends Migrated into the CVS	%
2006–2011	5	962	48	4.99%
2012–2015	4	962	16	1.66%
2016–2018	3	962	4	0.42%
2019–2023	4	961	4	0.42%
2006–2023	16	3847	72	1.87%

**Table 2:** The location of tips, proximal ends, model and age of leads spontaneously migrated into the cardiovascular system.

Location of Tips of Leads Spontaneously Migrated into CVS			Model of Leads Migrated into CVS		
**Lead Tip Location**	**N**	**%**	**Lead Model**	**N**	**%**
Right Ventricle	41	56.94%	BP passive	37	51.39%
Right atrium	28	38.89%	UP passive	30	41.67%
Superior vena cava	1	1.39%	BP VDD passive	1	1.39%
Coronary sinus	1	1.39%	BP active	1	1.39%
Azygos vein	1	1.39%	ICD passive	3	4.17%
All	72	100.0%	All	72	100.0%
Location of proximal ends of leads spontaneously migrated into CVS			Dwell time of leads spontaneously migrated into CVS [months]		
**Proximal End Location**	**N**	**%**	Range		17.24–376.4
Subclavian vein	28	38.89%	Median		137.5
Right Ventricle	13	18.06%	(Q1–Q3)		(92.50–194.0)
Anonymous vein	11	15.28%			
Superior vena cava	10	13.89%			
Pulmonary artery	7	9.72%			
Right atrium	2	2.78%			
Hepatic vein	1	1.39%			
All	72	100.0%			

CVS—cardiovascular system, BP—bipolar, UP—unipolar, VDD—atrial sensing, ventricular sensing/pacing lead, ICD—implantable cardioverter defibrillator, and SD—standard deviation.

**Table 3:** Characteristics of study groups and main indications for lead extraction.

Patient-Related Risk Factors for Extraction Complexity, Major Complications and Indications for TLE	TLE with Extraction of Leads Spontaneously Migrated into CVS	TLE without Extraction of Leads Spontaneously Migrated into CVS
Number of patients/forms of presenting the results	N = 72 Median (Q1–Q3) N (%)	N = 3775 Median (Q1–Q3) N (%)
Patient age during TLE [years]	65.00 (55.00–74.50)	69.00 (60.00–77.00) *p* = 0.158
Patient age at first system implantation [years]	50.50 (41.50–61.00)	61.00 (51.00–70.00) *p* = 0.001
Female	27 (37.50)	1437 (38.07) *p* = 0.981
Ischaemic heart disease	27 (37.50)	2123 (56.24) *p* = 0.002
NYHA class III and IV	6 (8.33)	592 (15.68) *p* = 0.124
Left ventricular ejection fraction [%]	60.00 (47.50–62.00)	54.00 (36.50–60.00) *p* = 0.121
Charlson co-morbidity index [points]	3 (1–6)	4 (2–6) *p* = 0.059
Indications for TLE		
Systemic infection	21 (29.17)	811 (21.48) *p* = 0.154
Local (pocket) infection	1 (1.39)	364 (9.64) *p* = 0.029
Mechanical lead damage (electrical failure)	20 (27.78)	1022 (27.04) *p* = 1.000
Lead dysfunction (exit/entry block, dislodgement, perforation extracardiac pacing)	2 (2.78)	861 (22.81) *p* = 0.001
Change of pacing mode/upgrading, downgrading	1 (1.39)	249 (6.60) *p* = 0.125
Abandoned lead/prevention of abandonment (atrial fibrillation, redundant leads)	2 (2.78)	100 (2.65) *p* = 0.762
Threatening/potentially threatening lead (loops, free ends, left heart, LDTVD)	23 (31.94)	106 (2.81) *p* = 0.001
Other (MRI indications, cancer, painful pocket, loss of indications for pacing/ICD)	0 (0.00)	128 (3.39) *p* = 0.209
Re-establishing venous access (symptomatic occlusion, SVC syndrome, lead replacement/upgrading)	2 (2.78)	130 (3.44) *p* = 0.985

CVS—cardiovascular system, TLE—transvenous lead extraction, NYHA FC class—New York Heart Association functional class, LDTVD—lead derived tricuspid valve defect, MRI—magnetic resonance imaging, ICD—implantable cardioverter defibrillator, SVC—superior vena cava. Abandoned lead/prevention of abandonment (AF, redundant leads), threatening/potentially threatening lead (loops, free ends, left heart, LDTVD), other (MRI indications, cancer, painful pocket, loss of indications for pacing/ICD) and re-establishing venous access (symptomatic occlusion, SVC syndrome, lead replacement/upgrading).

**Table 4:** System-, history of pacing- and procedure-related risk factors for major complications and increased extraction complexity in the study groups.

System-Related Risk Factors for Extraction Complexity, Major Complications and Indications for TLE	TLE with Extraction of Leads Spontaneously Migrated into CVS	TLE without Extraction of Leads Spontaneously Migrated into CVS
Number of patients/forms of presenting the results	N = 72 Median (Q1–Q3) N (%)	N = 3775 Median (Q1–Q3) N (%)
System and history of pacing		
Longest lead implant duration before TLE (months)	144.0 (96.48–194.0)	86.04 (45.00–133.0) *p* < 0.001
Global lead dwell time (years) before TLE	22.63 (15.00–34.21)	12.00 (6.00–20.67) *p* < 0.001
Abandoned leads before TLE	49 (68.06)	368 (9.75) *p* < 0.001
Unnecessary (large) lead loop in the heart	39 (54.17)	143 (3.79) *p* < 0.001
Number of leads in the heart before TLE	2 (1–2)	2 (1–2) *p* < 0.001
≥4 leads in the heart before TLE	16 (22.22)	102 (2.70) *p* < 0.001
Leads on both sides of the chest before TLE	18 (25.00)	89 (2.36) *p* < 0.001
Number of procedures before lead extraction	3 (2–3)	2 (1–2) *p* < 0.001
Two or more CIED procedures before TLE	64 (88.89)	1969 (52.16) *p* < 0.001
HV leads	12 (16,70)	1113 (29,50) *p* = 0.025
Values of risk indicators for major complications or increased complexity of the procedure		
SAFETY score [points]	8.82 (4.10–11.96	4.10 (2.73–8.82) *p* < 0.001
SAFETY score (estimation of MC risk) [%]	1.78 (0.48–4.19)	0.48 (0.33–1.78) *p* < 0.001
EROS score [points]	2 (1–3)	1 (1–2) *p* < 0.001
MB score [points] [need for advanced tools]	3 (2–4)	3 (2–4) *p* < 0.001
LED index [points]	14 (9–18)	9 (5–13) *p* < 0.001
Advanced TLE (Mazzone) scale (1–4) [points]	2 (2–3)	2 (2–3) *p* = 0.368
LECOM score [points]	12.51 (9.43–15.90)	7.53 (4.80–10.93) *p* < 0.001
LECOM score [%]	38.20 (20.55–61.67)	13.16 (6.55–28.33) *p* < 0.001
CID-TLE points ≥ 2	30 (41.67)	288 (7.63) *p* < 0.001
Potential extraction-related risk factors for major complications and procedure complexity		
Number of extracted leads per patient	2 (1–3)	2 (1–2) *p* < 0.001
Extraction of abandoned lead(s) (any)	47 (65.28)	336 (8.90) *p* < 0.001
Extraction of old model UP lead (excluding LV lead)	30 (41.67)	319 (8.45) *p* < 0.001
Extraction of passive fixation lead (excluding LV lead)	70 (97.22)	2160 (57.22) *p* < 0.001
Extraction of VDD lead	2 (2.78)	101 (2.68) *p* = 0.753
Extraction of leads with abnormal loop in the heart	38 (52.78)	146 (3.87) *p* < 0.001
Longest duration of extracted lead per patient (months)	137.5 (92.50–194.0)	84.00 (44.00–132.0) *p* < 0.001
Average duration time of extracted lead [years]	9.96 (6.67–15.50)	6.86 (3.50–10.67) *p* < 0.001

CVS—cardiovascular system, TLE—transvenous lead extraction, CIED—cardiac implantable electronic devices, SAFETY score (estimation of risk of major complications), MC—major complications, EROS score (risk of MC), MB score (need for advanced tools), LED index (predicted procedure fluoroscopy time), Advanced TLE (ALET—Mazzone—need for advanced TLE techniques), LECOM score (predicted procedure complexity), CID-TLE—complex indicator of difficulty of TLE procedure, ICD—implantable cardioverter defibrillator, LV—left ventricular, UP—unipolar, LV lead—left ventricular lead, and VDD—ventricular pacing/sensing, atrial sensing lead.

**Table 5:** Procedure complexity, complications, and long-term outcome in the study groups.

Procedure Complexity, Complications, and Long-Term Outcome	TLE with Extraction of Leads Spontaneously Migrated into CVS	TLE without Extraction of Leads Spontaneously Migrated into CVS
Number of patients/forms of presenting the results	N = 72 Median (Q1–Q3) N (%)	N = 3775 Median (Q1–Q3) N (%)
TLE complexity and outcomes		
Procedure duration (sheath-to-sheath) [minutes]	55.00 (55.00–87.50)	9.00 (4.00–12.00) *p* < 0.001
Time of single lead extraction (sheath-to-sheath/number of extracted leads) [minutes]	50.00 (27.50–55.00)	4.50 (4.00–9.00) *p* < 0.001
Total number of patients with technical problems	57 (79.17)	672 (17.80) *p* < 0.001
Number of technical problems per patient	1 (1–2)	0 (0–0) *p* < 0.001
Two or more Technical Problems	18 (25.00)	216 (5.72) *p* < 0.001
Use of additional tools		
Evolution (old and R-L), TightRail or metal sheaths	6 (8.33)	372 (9.86) *p* = 0.818
Lasso catheters/snares	38 (52.78)	111 (2.94) *p* < 0.001
Basket catheter	33 (45.83)	22 (0.58) *p* < 0.001
Use of loops	51 (70.83)	4 (0.11) *p* < 0.001
Use of pig-tail catheters	8 (11.11)	16 (0.42) *p* < 0.001
Alternative approach	57 (79.17)	70 (1.85) *p* = 0.003
CID-TLE score [points]	4 (4–4)	0 (0–0) *p* < 0.001
CID-TLE score: 3 and more points	70 (97.22)	294 (7.79) *p* < 0.001
TLE efficacy and complications		
Major complications (any)	2 (2.78)	76 (2.01) *p* = 0.973
Haemopericardium	1 (1.39)	45 (1.19) *p* = 0.693
Haemothorax	0 (0.00)	5 (0.13) *p* = 0.180
Tricuspid valve damage during TLE (severe)	1 (1.39)	22 (0.58) *p* = 0.915
Rescue cardiac surgery	1 (1.39)	39 (1.03) *p* = 0.771
Death procedure-related (intra- and postprocedural)	1 (1.39)	5 (0.13) *p* = 0.243
Clinical success	66 (91.67)	3703 (98.09) *p* < 0.001
Procedural success	63 (87.50)	3601 (95.39) *p* < 0.001
Radiographic success		
Success	63 (87.50)	3620 (95.89) *p* < 0.001
Retained tip of lead	5 (6.94)	74 (1.96) *p* = 0.012
Retained short lead fragment	2 (2.78)	65 (1.72) *p* = 0.823
Retained long lead fragment	0 (0.00)	6 (0.16) *p* = 0.243
Lead remnant removal during emergency or planned cardiac surgery	2 (2.78)	4 (0.11) *p* <0.001
Mortality after TLE		
1-year mortality	9 (12.50)	304 (8.05) Chi^2^; *p* = 0.250 Log rank; *p* = 0.148
3-year mortality	11 (15.28)	649 (17.19) Chi^2^; *p* = 0.788 Log rank *p* = 0.834
All deaths	28 (38.89)	1326 (35.13) Chi^2^ *p* = 0.591 Log rank *p* = 0.241

CVS—cardiovascular system, TLE—transvenous lead extraction, and CID-TLE—complex indicator of difficulty of TLE procedure.

**Table 6:** Factors affecting migration of the proximal lead end into the cardiovascular system. Results of univariable and multivariable regression analysis.

	Univariable Regression			Multivariable Regression		
	OR	95%CI	*p*	OR	95%CI	*p*
Patient age at first CIED [by 1 year]	0.981	0.970–0.992	0.008	0.996	0.977–1.016	0.690
Ischaemic heart disease	0.484	0.301–0.779	0.003	0.917	0.511–1.644	0.770
NYHA FC class [by 1]	0.368	0.247–0.550	*p* < 0.001	0.380	0.221–0.653	*p* < 0.001
Left ventricular ejection fraction [by 1% *p*]	1.016	1.000–1.032	0.051	0.982	0.959–1.005	0.130
Charlson Co-morbidity Index [by 1 point]	0.917	0.853–0.986	0.019	0.998	0.911–1.094	0.972
Dwell time of the oldest extracted lead before TLE [by 1 year]	1.079	1.049–1.109	*p* < 0.001	1.010	0.962–1.060	0.694
Abandoned lead [yes/no]	0.917	0.853–0.986	0.019	8.473	4.270–16.81	*p* < 0.001
Number of leads before TLE [by 1]	1.038	0.999–1.097	0.054	1.226	0.863–1.744	0.255
Leads on both sides of the chest	14.30	8.148–2.100	*p* < 0.001	1.981	1.015–3.867	0.045
Number of previous CIED-related procedures [by one]	1.045	1.005–1.087	0.027	0.997	0.722–1.377	0.985
HV lead presence	0.468	0.251–0.871	0.017	0.722	0.342–1.524	0.393

NYHA FC class—New York Heart Association functional class, %—percent point, TLE—transvenous lead extraction, CIED—cardiac implantable electronic devices, and HV lead—high voltage (defibrillator lead).

## Data Availability

Data are available upon request to authors.
